# Supplementary material for: Isogenic Cell Lines Derived from Specific Organ Metastases Exhibit Divergent Cytogenomic Aberrations
Source: Cancers (Basel). 2023 Feb 23;15(5):1420. doi: 10.3390/cancers15051420 (PMC10000985; doi:10.3390/cancers15051420)

Table S1. Relative to the 1° tumor cell line, fold change (F.C. > -1.5 & ≤ -1.25) of proteins in metastatic cell lines that if dysregulated contribute to CIN/aneuploidy and are then impacted by CIN/aneuploidy.

Biological Process	Protein	Description	Chr Location	F.C. Brain	F.C. Liver	F.C. Lung	F.C. Spine
Cell Cycle Regulation	ATAD2	ATPase family, AAA domain containing 2	8q24.13	-1.31			
	CCNB1	Cyclin B1	5q13.2			-1.30	-1.26
	CDC25B	Cell division cycle 25B	20p13	-1.34			
	CDCA3	Cell division cycle associated 3	12p13.31			-1.39	
	CDK1	Cyclin-dependent kinase 1	10q21.2			-1.26	
	CDS1B	CDC28 protein kinase regulatory subunit 1B	1q21.3		-1.37		
	FAM83D	Family with sequence similarity 83 member D	20q11.23	-1.43			
	PA2G4	Proliferation-associated 2G4	12q13.2			-1.30	
	PBK	PDZ binding kinase	8p21.1	-1.29			
	PRR11	Proline rich 11	17q22		-1.34		
	UBEC2	Ubiquitin conjugating enzyme E2C	20q13.12		-1.28	-1.25	
	UBE2S	Ubiquitin conjugating enzyme E2S	19q13.42	-1.47			
	WEE1	WEE1 G2 checkpoint kinase	11p15.4	-1.27	-1.30		
	AURKA	Aurora kinase A	20q13.2	-1.29			
	DTL	Denticleless E3 ubiquitin protein ligase homolog (Drosophila)	1q32.3	-1.32			
	GTSE1	G2 and S-phase expressed 1	22q13.31	-1.47			
	PLK1	Polo-like kinase 1	16p12.2	-1.34			
Centrosome Regulation	CCT3	Chaperonin containing TCP1, subunit 3 gamma	1q22		-1.40		
	CEP152	Centrosomal protein 152kDa	15q21.1	-1.42			
	CEP192	Centrosomal protein 192kDa	18p11.21				-1.49
	CEP72	Centrosomal protein 72kDa	5p15.33		-1.28		
	KIF24	Kinesin family member 24	9p13.3				-1.24
Cytokinesis	ASPM	Abnormal spindle microtubule assembly	1q31.3			-1.30	
	CEP55	Centrosomal protein 55kDa	10q23.33	-1.33			
	CIT	Citron Rho-interacting serine/threonine kinase	12q24.23	-1.45			
	KIF20A	Kinesin family member 20A	5q31.2	-1.30			
	KIF20B	Kinesin family member 20B	10q23.31	-1.27			
	PRC1	Protein regulator of cytokinesis 1	15q26.1			-1.37	
	INCENP	Inner centromere protein	11q12.3	-1.27			
	ECT2	Epithelial cell transforming 2	3q26.31	-1.35			
Chromosome Partition	BUB1	BUB1, mitotic checkpoint serine/threonine kinase	2q13	-1.30			
	CDCA8	Cell division cycle associated 8	1p34.3	-1.30			
	ESPL1	Extra spindle pole bodies like 1, separase	12q13.13	-1.47			
	KNL1	Kinetochore scafford 1	15q15.1	-1.25			
	MIS18BP1	MIS 18 binding protein 1	14q21.2		-1.33		
	SGO1	Shugoshin 1	3p24.3	-1.28			
	CDCA2	Cell division cycle associated 2	8p21.2	-1.32			
DNA Condensation	ESCO2	Establishment of sister chromatid cohesion N-acetyltransferase 2	8q21.1		-1.27		
	NCAPD3	Non-SMC condensing II complex subunit D3	11q25	-1.36			

	NCAPG	Non-SMC condensing I complex subunit G	4p15.31			-1.31	
	NCAPH	Non-SMC condensing I complex subunit H	2q11.2	-1.40	-1.27		
	SMC2	Structural maintenance of chromosomes 2	9q31.1	-1.46			-1.32
Kinetochore Formation	CENPI	Centromere protein I	Xq22.1	-1.49	-1.49		
	SPDL1	Spindle apparatus coiled-coil protein 1	5q35.1			-1.30	
	NDC80	NDC80 kinetochore complex component	18p11.32				
	KNTC1	Kinetochore associated 1	12q24.31	-1.25		-1.36	
Microtubule Regulation	KIF15	Diaphanous related formin 3	13q21.2	-1.29			
	KIF18B	Kinesin family member 4A	Xq13.1	-1.43			
	SKA3	Spindle and kinetochore associated complex subunit 3	13q12.11			-1.39	
	PCNT	Pericentrin	21q22.3	-1.32			
	STMN1	Stathmin 1	1q36.11			-1.56	
Nuclear Envelope Regulation	NUP85	Nucleoporin 85kDa	17q25.1		-1.43	-1.37	
	NUP93	Nucleoporin 93kDa	16p13	-1.48	-1.49		-1.29
	SUGT1	SGT1 homolog, MIS12 kinetochore complex assembly cochaperone	13q14.3			-1.32	
	TMPO	Thymopoietin	12q23.1			-1.30	
	VRK1	Vaccinia related kinase 1	14q32.2	-1.27			
Spindle Assembly & Regulation	HASPIN	Histone H3 associated protein kinase	17p13.2		-1.29		
	KIF11	Kinesin family member 11	10q23.33	-1.28			
	KPNB1	Karyopherin (importin) beta 1	17q21.32	-1.29	-1.353		-1.40
	WDR62	WD repeat domain 62	19q13.12			-1.28	
	HAUS7	HAUS augmin like complex subunit 7	Xq28		-1.28		
Spindle Checkpoint	ANAPC1	Anaphase promoting complex subunit 1	2q13	-1.330			
	BUB1B	Mitotic checkpoint serine/threonine kinase B	15q15.1	-1.27			
	CDC20	Cell division cycle 20	1p34.2	-1.35			
	ERCC6L	Excision repair cross-complementation group 6-like	Xq13.1	-1.29			
	TRIP13	Thyroid hormone receptor interacter 13	5p15.33				-1.36
	ZW10	ZW10 kinetochore protein	11q23.2				-1.45
	ZWILCH	ZWILCH kinetochore protein	15q22.31		-1.31	-1.46	
DNA Damage	ATPAD5	ATPase family, AAA domain containing 5	17q11.2	-1.33			
	DONSON	DNA damage-induced apoptosis suppressor	21q22.11	-1.25	-1.47		
	MTBP	MDM2 binding protein	8q24.12			-1.36	
	PARP2	Poly(ADP-ribose) polymerase 2	14q11.2		-1.34		
	RAD18	RAD18, E3 ubiquitin protein ligase	3p25.3				
	RUVBL2	RuvB-like AAA ATPase 2	19q13.33		-1.32		
	TTI1	TELO2 interacting protein 1	20q11.23	-1.35			-1.49
	PCLAF	PCNA clamp associated factor	15q22.31		-1.34		
	DDX11	DEAD/H (Asp-Glu-Ala-Asp/His) box helicase 11	12p11.21				
	LIG1	Ligase I, DNA, ATP-dependent	19q13.33	-1.29			
	PARP1	Poly(ADP-ribose) polymerase 1	1q42.12	-1.36			

	RPA3	Replication protein A3	7p21.3		-1.36		
	POLE	Polymerase (DNA directed), epsilon catalytic subunit	12q24.33		-1.38		
	CHEK2	Checkpoint kinase 2	22q12.1			-1.28	
	TIPIN	TIMELESS interacting protein	15q22.31	-1.40			
	PCNA	Proliferating cell nuclear antigen	20p12.3	-1.38			
	FIGNL1	Fidgetin-like 1	7p12.2	-1.47			
	HMGB1	High mobility group box 1	13q12.3			-1.34	
	HMGB2	High mobility group box 2	4q34.1			-1.44	
	NUCKS 1	Nuclear casein kinase & cyclin-dependent kinase substrate 1	1q32.1		-1.39		
	RAD54B	RAD54 homolog B (S. cerevisiae)	8q22.1	-1.38			
	CHEK1	Checkpoint kinase 1	11q24.2				
	BRACA2	Breast cancer 2	13q13.1	-1.45			
	ATRIP	ATR interacting protein	3p21.31				
	FANCD2	Fanconi anemia complementation group D2	3p25.3	-1.27			
	FANCI	Fanconi anemia complementation group I	15q26.1	-1.31		-1.27	
	USP1	Ubiquitin specific peptidase 1	1p31.3	-1.36			
	XRCC4	X-ray repair complementing defective repair in Chinese hamster cells 4	5q14.3			-1.34	
	XRCC6	X-ray repair complementing defective repair in Chinese hamster cells 6	22q13.2			-1.47	-1.34
DNA Replication	RMI1	RecQ mediated genome instability 1	9q21.32		-1.26		
	POLA1	Polymerase (DNA directed), alpha 1, catalytic subunit	Xp22.11-p21.3	-1.41			
	POLE2	Polymerase (DNA directed), epsilon 2, accessory subunit	14q21.3		-1.26	-1.35	
	POLE3	Polymerase (DNA directed), epsilon 3, accessory subunit	9q32		-1.38		
	RFC3	Replication factor C sunuint 3	13q13.2				-1.266
	RFC5	Replication factor C subunit 5	12q24.23		-1.46		
	TOPBP1	Topoisomerase (DNA) II binding protein 1	3q22.1	-1.26			
	WRAP53	WD repeat containing, antisense to TP53	17p13.3	-1.38			
DNA Metabolism	DCK	Deoxycytidine kinase	4q13.3	-1.33			
	DCTPP1	dCTP pyrophosphatase 1	16p11.2	-1.36		-1.45	
	DHFR	Dihydrofolate reductase	5q14.1			-1.38	
	DTYMK	Deoxythymidylate kinase	2q37.3	-1.25	-1.38	-1.29	
	DUT	Deoxyuridine triphosphatase	15q21.1	-1.47	-1.31		
	PRPS2	Phosphoribosyl pyrophosphate synthetase 2	Xp22.2		-1.30		
	RRM2	Ribonucleotide reductase M2	2q25.1		-1.36		
	SLC29A1	Solute carrier family 29 (equilibrative nucleoside transporter), member 1	6p21.1		-1.36		
	TK1	Thymidine kinase 1, soluble	17q25.3		-1.34		-1.25
	TYMS	Thymidylate synthetase	18p11.32		-1.49	-	
Chromatin Organization	ANP32B	Acidic nuclear phosphoprotein 32 family member B	9q22.33		-1.38		
	HIST1H1C	Histone cluster 1, H1c	6p22.2		-1.26		
	HIST1H4A	Histone cluster 1, H4a	6p22.2		-1.43		

HIST1H4B	Histone cluster 1, H4b	6p22.2	-1.43
HIST1H4C	Histone cluster 1, H4c	6p22.2	-1.43
HIST1H4D	Histone cluster 1, H4d	6p22.2	-1.43
HIST1H4E	Histone cluster 1, H4e	6p22.2	-1.43
HIST1H4F	Histone cluster 1, H4f	6p22.2	-1.43
HIST1H4I	Histone cluster 1, H4i	6p22.2	-1.43
HIST1H4J	Histone cluster 1, H4j	6p22.2	-1.43
HIST1H4K	Histone cluster 1, H4k	6p22.2	-1.43
HIST1H4L	Histone cluster 1, H4l	6p22.2	-1.43
HIST2H4B	Histone cluster 2, H4b	1q21.2	-1.43

Yellow-beige shadings indicates that no gene counterparts were observed for the proteins in these processes.

Table S2. Relative to the 1° tumor cell line, fold change (F.C. ≥ 1.25) of proteins in metastatic cell lines that if dysregulated contribute to CIN/aneuploidy and are then impacted by CIN/aneuploidy.

Biological Process	Protein	Description	Chr Location	F.C. Brain	F.C. Liver	F.C. Lung	F.C. Spine
Centrosome Regulation	CEP152	Centrosomal protein 152kDa	15q21.1		1.30	1.27	1.48
	CEP41	Centrosomal protein 41kDa	7q32.2			1.27	
	Kif24	Kinesin family member 24	9p13.3			1.43	
	SASS6	SAS-6 centriolar assembly protein	1p21.2				1.31
Kinetochore Formation	CENPU	Centromere protein U	4q35.1			1.31	1.50
	MIS18A	MIS18 kinetochore protein A	1q41	1.86			1.42
Microtubule Regulation	STATHN1	Stathmin 1	1p36.11	1.28			
DNA Damage	PARP2	Poly(ADP-ribose) polymerase 2	14q11.2	1.37			1.29
	HMGB1	High mobility group box 1	13q12.3	1.29			
	HMGB2	High mobility group box 2	4q34.1	1.53			
	INC	INTS3 and NABP interacting protein	9q32	1.34	1.84	1.25	
	NSMCE4A	NSE4 homolog A, SMC5-SMC6 complex component	10q26.13	1.27			
	NUCKS1	Nuclear casein kinase & cyclin-dependent kinase substrate 1	1q32.1				1.28
	XRCC1	X-ray repair complementing defective repair in Chinese hamster cells 1	19q13.31			1.25	
	MDC1	Mediator of DNA damage checkpoint 1	6p21.33			1.47	
DNA Replication	NASP	Nuclear autoantigenic sperm protein (histone-binding)	1p34.1	1.32			
	CDC6	Cell division cycle 6	17q21.2			1.44	1.82
	ORC1	Origin recognition complex subunit 1	1p23.3	1.36	1.36		
	ORC2	Origin recognition complex subunit 2	2q33.1	1.32			
	DBF4	DBF4 zinc finger	7q21.12				1.41
Chromatin Organization	HIST1H1B	Histone cluster 1, H1b	6p22.1				1.31
	HIST1H1C	Histone cluster 1, H1c	6p22.2			1.28	1.27
	HIST1H2AC	Histone cluster 1, H2ac	6p22.2		1.29	1.25	1.27
	HIST1H3A	Histone cluster 1, H3a	6p22.2	1.48	1.25		
	HIST1H3B	Histone cluster 1, H3b	6p22.2	1.48	1.25		
	HIST1H3C	Histone cluster 1, H3c	6p22.2	1.48	1.25		

HIST1H3D	Histone cluster 1, H3d	6p22.2	1.48	1.25		
HIST1H3F	Histone cluster 1, H13f	6p22.2	1.48	1.25		
HIST1H3G	Histone cluster 1, H3g	6p22.2	1.48	1.25		
HIST1H3I	Histone cluster 1, H3i	6p22.2	1.48	1.25		
HIST1H3J	Histone cluster 1, H3j	6p22.2	1.48	1.25		
HIST1H4A	Histone cluster 1, H4a	6p22.2	1.40			
HIST1H4B	Histone cluster 1, H4b	6p22.2	1.40			
HIST1H4C	Histone cluster 1, H4c	6p22.2	1.40			
HIST1H4D	Histone cluster 1, H4d	6p22.2	1.40			
HIST1H4E	Histone cluster 1, H4e	6p22.2	1.40			
HIST1H4F	Histone cluster 1, H4f	6p22.2	1.40			
HIST1H4I	Histone cluster 1, H4i	6p22.1	1.40			
HIST1H4J	Histone cluster 1, H4j	6p22.1	1.40			
HIST1H4K	Histone cluster 1, H4k	6p22.1	1.40			
HIST1H4L	Histone cluster 1, H4l	6p22.1	1.40			
HIST2H2AA4	Histone cluster 2, H2AA4	1q21.2		1.78		1.25
HIST2H4B	Histone cluster 2, H4b	1q21.2	1.40			

Yellow-beige shadings indicates that no gene counterparts were observed for the proteins in these processes.

Supplemental Materials

Figures

Paul T Winnard Jr[1], Laura Morsberger[2], Raluca Yonescu[2], Liqun Jiang[2], Ying S. Zou[2]*, Venu Raman[1,3,4,5]*

[1] Department of Radiology and Radiological Sciences, Johns Hopkins University School of Medicine, Baltimore, MD, USA

[2]Department of Pathology, Johns Hopkins University School of Medicine, Baltimore, MD, USA

[3]Department of Oncology, Johns Hopkins University School of Medicine, Baltimore, MD, USA

[4]Pharmacology and Molecular Sciences, Johns Hopkins School of Medicine, Baltimore, MD, USA

[5]Department of Pathology, University Medical Center Utrecht, Utrecht, The Netherlands

Supplemental Figure Legends

Supplemental Figure S1. Comparisons of brain cell line karyotypes to lung and spine cell line karyotypes as well as a comparison of lung cell line karyotypes to spine cell line karyotypes. Detailed modal descriptions of the comparisons are presented here. In all three comparisons distinctions in karyotypes are highlighted. Inter-karyotype heterogeneity between the 1° tumor cell line karyotype (see Figures 2 and 3) and the liver, lung, and spine cell line karyotypes are indicated by blue arrows. Intra-karyotype heterogeneity is indicated by orange arrows for the 5 brain (Br-1 – Br-5) cell line karyotypes, green arrows for the 3 lung (Lu-1 – Lu-3) cell line karyotypes, and a red arrow for the 2 spine (Sp-1 & Sp-2) cell line karyotypes.

Modal brain cell line karyotype: 56,XX,+1,der(1;7)(q10;q10),add(1)(q21),+2,add(3)(p25),add(3)(p14),+add(3)(q12),+5,-6, add(6)(q13)x2,+dup(6)(p21.3p22),+7,i(7)(q10),-8,-8,+9,inv(9)(q13q?22),+11,add(11)(p14),add(11)(p14),del(12)(p12),-13,-13,-14, +15,add(15)(p11.2),add(18)(p11.2),-19,add(19)(p13),add(20)(q13.2)x2,-21,-22,+mar1,+mar2,+mar3,+mar4,+mar6,+mar10,+mar12,+mar13,+mar
Compared to:
Modal lung cell line karyotype: 55,XX,+1,der(1;7)(q10;q10),add(1)(q21),+2,add(3)(p25),add(3)(p14),+add(3)(q12),+4,+5,dup(6)(p21.3p22),i(7)(q10),-8,-8, +9,inv(9)(q13q?22),+11,add(11)(p14),add(11)(p14),del(12)(p12),-13,-13,-14,add(15)(p11.2),add(18)(p11.2),-19,add(19)(p13), add(20(q13.2)x2,-21,-22,+mar1,+mar2,+mar3,+mar4,+mar5,+mar6,+mar7,+mar8,+mar11,+mar

Modal brain cell line karyotype: 56,XX,+1,der(1;7)(q10;q10),add(1)(q21),+2,add(3)(p25),add(3)(p14),+add(3)(q12),+5,-6, add(6)(q13)x2,+dup(6)(p21.3p22),+7,i(7)(q10),-8,-8,+9,inv(9)(q13q?22),+11,add(11)(p14),add(11)(p14),del(12)(p12),-13,-13,-14, +15,add(15)(p11.2),add(18)(p11.2),-19,add(19)(p13),add(20)(q13.2)x2,-21,-22,+mar1,+mar2,+mar3,+mar4,+mar6, +mar10,+mar12,+mar13,+mar
Compared to:
Modal spine cell line karyotype: 56,XX,+1,der(1;7)(q10;q10),add(1)(q21),+2,add(3)(p25),add(3)(p14),+5,add(6)(q13)x2,+dup(6)(p21.3p22),+7,i(7)(q10),-8,-8,+9,inv(9)(q13q?22),+11,add(11)(p14),add(11)(p14),del(12)(p12),-13,-13,-14,+15,add(15)(p11.2),add(18)(p11.2),-19, add(19)(p13),add(20)(q13.2)x2,-21,-22,+mar1,+mar2,+mar3,+mar6,+mar7,+mar10,+mar12,+mar13,+2mar

Modal lung cell line karyotype: 55,XX,+1,der(1;7)(q10;q10),add(1)(q21),+2,add(3)(p25),add(3)(p14),+add(3)(q12),+4,+5,dup(6)(p21.3p22),i(7)(q10),-8,-8,+9,inv(9)(q13q?22),+11,add(11)(p14),add(11)(p14),del(12)(p12),-13,-13,-14,add(15)(p11.2),add(18)(p11.2),-19, add(19)(p13),add(20(q13.2)x2,-21,-22,+mar1,+mar2,+mar3,+mar4,+mar5,+mar6,+mar7,+mar8,+mar11,+mar
Compared to:
Modal spine cell line karyotype: 56,XX,+1,der(1;7)(q10;q10),add(1)(q21),+2,add(3)(p25),add(3)(p14),+5,add(6)(q13)x2, +dup(6)(p21.3p22),+7,i(7)(q10),-8,-8,+9,inv(9)(q13q?22),+11,add(11)(p14),add(11)(p14),del(12)(p12),-13,-13,-14,+15, add(15)(p11.2),add(18)(p11.2),-19,add(19)(p13),add(20)(q13.2)x2,-21,-22,+mar1,+mar2,+mar3,+mar6,+mar7,+mar10, +mar12,+mar13,+2mar

Supplemental Figure Legends

Supplemental Figure S2. Comparisons of liver cell line karyotypes to lung and spine cell line karyotypes. Detailed modal descriptions of the comparisons are presented here. In the two comparisons distinctions in karyotypes are highlighted. Inter-karyotype heterogeneity between the 1º tumor cell line karyotype (see Figures 2 and 3) and the liver, lung, and spine cell line karyotypes are indicated by blue arrows. Intra-karyotype heterogeneity is indicated by violet arrows for the 5 liver (Li-1 – Li-5) cell line karyotypes, green arrows for the 3 lung (Lu-1 – Lu-3) cell line karyotypes, and a red arrow for the 2 spine (Sp-1 & Sp-2) cell line karyotypes.

Modal liver cell line karyotype: 56,XX,+1,der(1;7)(q10;q10),add(1)(q21),+2,add(3)(p25),add(3)(p14),+add(3)(q12),+5,-6, dup(6)(p21.3p22),+7,i(7)(q10),-8,-8,+9,inv(9)(q13q?22),-10,+11,add(11)(p14),add(11)(p14),del(12)(p12),-13,-13,-14,+15, add(15)(p11.2),add(18)(p11.2),-19,add(19)(p13),add(20)(q13.2)x2,-21,-22,+mar1,+mar3,+mar4,+mar6,+mar7,+mar8, +mar11,+mar12,+mar13,+3mar
Compared to:
Modal lung cell line karyotype: 55,XX,+1,der(1;7)(q10;q10),add(1)(q21),+2,add(3)(p25),add(3)(p14),+add(3)(q12),+4,+5,dup(6)(p21.3p22),i(7)(q10),-8,-8,+9, inv(9)(q13q?22),+11,add(11)(p14),add(11)(p14),del(12)(p12),-13,-13,-14,add(15)(p11.2),add(18)(p11.2),-19,add(19)(p13), add(20(q13.2)x2,-21,-22,+mar1,+mar2,+msr3,+mar4,+mar5,+mar6,+mar7,+mar8,+mar11,+mar

Modal liver cell line karyotype: 56,XX,+1,der(1;7)(q10;q10),add(1)(q21),+2,add(3)(p25),add(3)(p14),+add(3)(q12),+5,-6, dup(6)(p21.3p22),+7,i(7)(q10),-8,-8,+9,inv(9)(q13q?22),-10,+11,add(11)(p14),add(11)(p14),del(12)(p12),-13,-13,-14,+15, add(15)(p11.2),add(18)(p11.2),-19,add(19)(p13),add(20)(q13.2)x2,-21,-22,+mar1,+mar3,+mar4,+mar6,+mar7,+mar8,+mar11, +mar12,+mar13,+3mar
Compared to:
Modal spine cell line karyotype: 56,XX,+1,der(1;7)(q10;q10),add(1)(q21),+2,add(3)(p25),add(3)(p14),+5,add(6)(q13)x2, +dup(6)(p21.3p22),+7,i(7)(q10),-8,-8,+9,inv(9)(q13q?22),+11,add(11)(p14),add(11)(p14),del(12)(p12),-13,-13,-14,+15, add(15)(p11.2),add(18)(p11.2),-19,add(19)(p13),add(20)(q13.2)x2,-21,-22,+mar1,+mar2,+mar3,+mar6,+mar7,+mar10,+mar12, +mar13,+2mar

Supplemental Figure S1

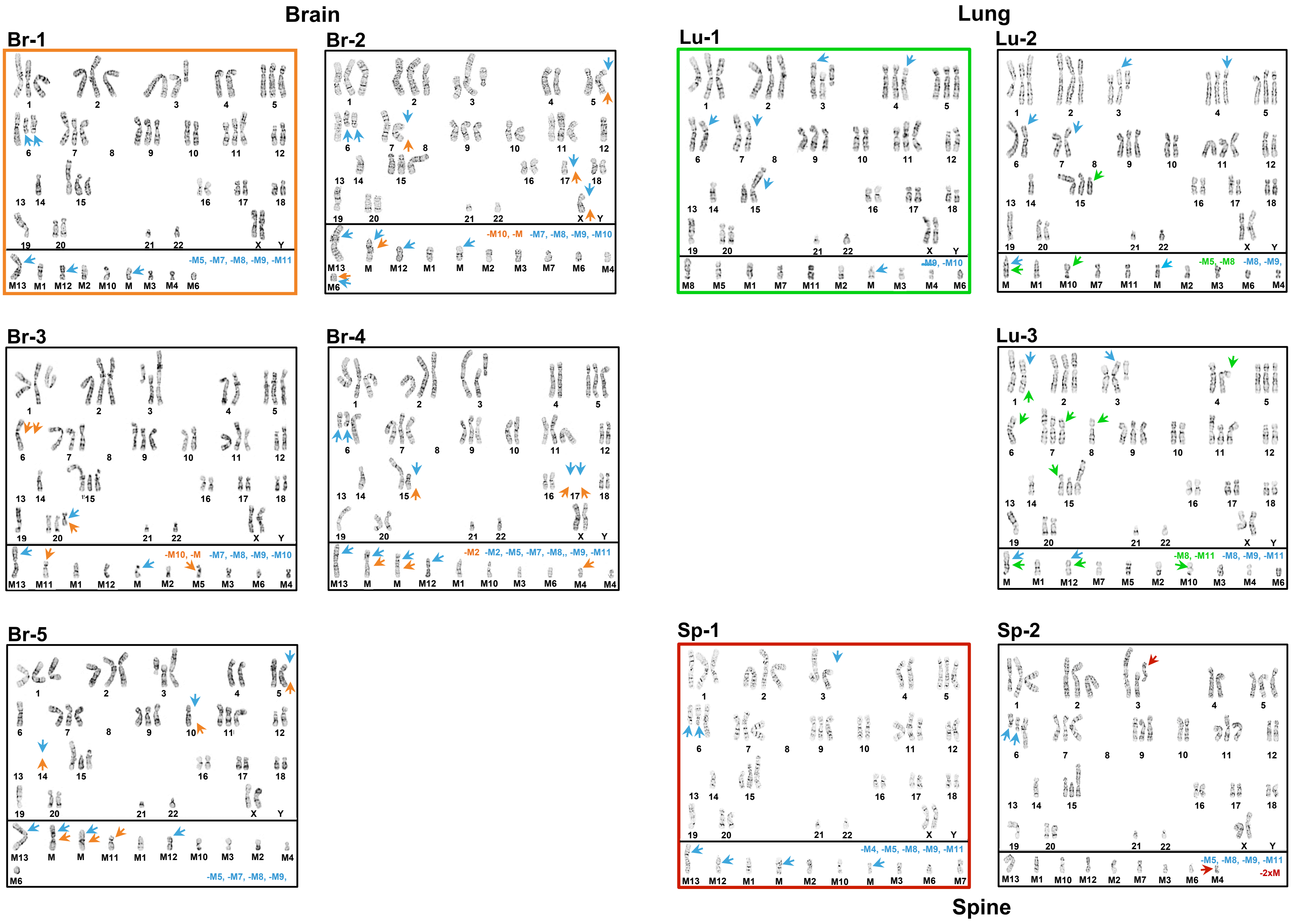

Supplemental Figure S2

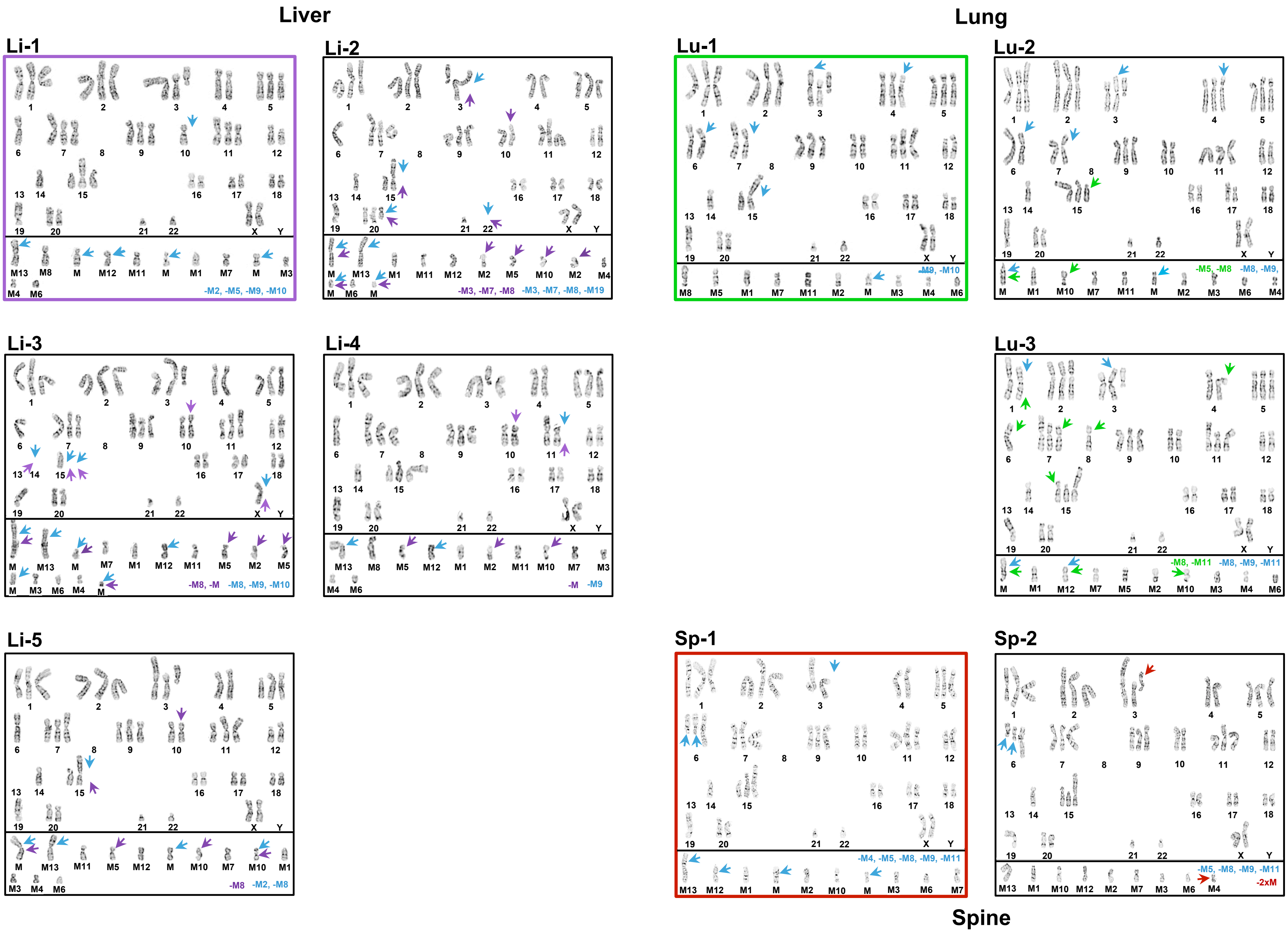

Supplement: Supplementary file 1 [file cancers-15-01420-s001.zip › cancers-2208674-supplementary.pdf]
